# An Observational Cohort Study of *Clostridium difficile* Ribotype 027 and Recurrent Infection

**DOI:** 10.1128/mSphere.00033-18

**Published:** 2018-05-23

**Authors:** Krishna Rao, Peter D. R. Higgins, Vincent B. Young

**Affiliations:** a Department of Internal Medicine, University of Michigan, Ann Arbor, Michigan, USA; b Division of Infectious Diseases, University of Michigan, Ann Arbor, Michigan, USA; c Division of Gastroenterology, University of Michigan, Ann Arbor, Michigan, USA; d Department of Microbiology and Immunology, University of Michigan, Ann Arbor, Michigan, USA; Centers for Disease Control and Prevention

**Keywords:** *Clostridium difficile*, biomarkers, clinical decision making, molecular epidemiology, ribotyping

## Abstract

Recurrent Clostridium difficile infection (rCDI) frequently complicates recovery from CDI. Accurately predicting rCDI would allow judicious allocation of limited resources, but published models have met with limited success. Thus, biomarkers predictive of recurrence have been sought. This study tested whether PCR ribotype independently predicted rCDI. Stool samples from nonpregnant inpatients ≥18 years of age with diarrhea were included from October 2010 to January 2013 after the patients tested positive for C. difficile in the clinical microbiology laboratory. Per guidelines, the rCDI was defined as a positive test for C. difficile at >2 weeks but ≤8 weeks from the index episode. For each sample, a single colony of C. difficile was isolated by anaerobic culture, confirmed to be toxigenic by PCR, and ribotyped. Simple logistic regression and multiple logistic regression were used to model the primary outcome of rCDI, incorporating a wide range of clinical parameters. In total, 927 patients with 968 index episodes of CDI were included, with 110 (11.4%) developing rCDI. Age and use of proton pump inhibitors or concurrent antibiotics did not increase the risk of rCDI. Low serum bilirubin levels and ribotype 027 were associated with increased risk of rCDI on unadjusted analysis, with health care-associated CDI being inversely associated. In the final multivariable model, ribotype 027 was the strongest independent predictor of rCDI (odds ratio, 2.17; 95% confidence interval, 1.33 to 3.56; *P* = 0.002). Ribotype 027 is an independent predictor of rCDI.

**IMPORTANCE** CDI is a major public health issue, with over 400,000 cases per year in the United States alone. Recurrent CDI is common, occurring in approximately one in five individuals after a primary episode. Although interventions exist that could reduce the risk of recurrence, deployment in all patients is limited by cost, invasiveness, and/or an undetermined long-term safety profile. Thus, clinicians need risk stratification tools to properly allocate treatments. Because prior research on clinical predictors has failed to yield a reliable, reproducible, and effective predictive model to assist treatment decisions, accurate biomarkers of recurrence would be of great value. This study tested whether PCR ribotype independently predicted rCDI, and the data build upon prior research in showing that ribotype 027 is associated with rCDI.

## Abstract (precis)

CDI is a major public health issue, with over 400,000 cases per year in the United States alone. Recurrent CDI is common, occurring in approximately one in five individuals after a primary episode. Although interventions exist that could reduce the risk of recurrence, deployment in all patients is limited by cost, invasiveness, and/or an undetermined long-term safety profile. Thus, clinicians need risk stratification tools to properly allocate treatments. Because prior research on clinical predictors has failed to yield a reliable, reproducible, and effective predictive model to assist treatment decisions, accurate biomarkers of recurrence would be of great value. This study tested whether PCR ribotype independently predicted rCDI, and the data build upon prior research in showing that ribotype 027 is associated with rCDI.

## INTRODUCTION

Clostridium difficile infection (CDI) is responsible for over 400,000 cases of infectious colitis and over 30,000 deaths per year in the United States alone (1). Even among those who recover, recurrent CDI is common and affects approximately 20% of patients, many of whom are readmitted or have further recurrences (1). The estimated cost of recurrent CDI alone in the United States is up to $2.8 billion annually (2). Although newer therapies that reduce the risk of recurrent CDI, such as the use of fidaxomicin (3), monoclonal antibodies (4), and fecal microbiota transplantation (FMT) (5, 6), are available, their widespread deployment in all patients is limited by cost (7) and/or undetermined safety profiles (8). Thus, clinicians are in need of tools to achieve stratification of patients for risk of recurrence and consequently to better allocate limited resources.

Models utilizing clinical variables alone to predict recurrent CDI in patients presenting with an index episode have been developed (9–11). However, when validation of these models in external cohorts was attempted, they failed to make accurate predictions (12). There is evidence that biomarkers based on the immune response (13–15), the microbiota (16, 17), or the infecting strain (18–22) are associated with recurrence. The hope is that the use of such biomarkers will improve the predictive performance of clinical models. Here, in an observational cohort study, we tested the hypothesis that infection with specific C. difficile strains, as determined by the PCR ribotype, is associated with a greater risk of recurrence. We specifically focus on the ribotype 027 strain, given its importance in the hospital setting (23, 24), where our study took place, compared to the outpatient, community setting, where different strains may predominate (24).

(Parts of this work were previously presented at the Anaerobe 2016 conference in Nashville, TN, on 14 July 2016.)

## RESULTS

### Descriptive and unadjusted statistics.

**FIG 1:**
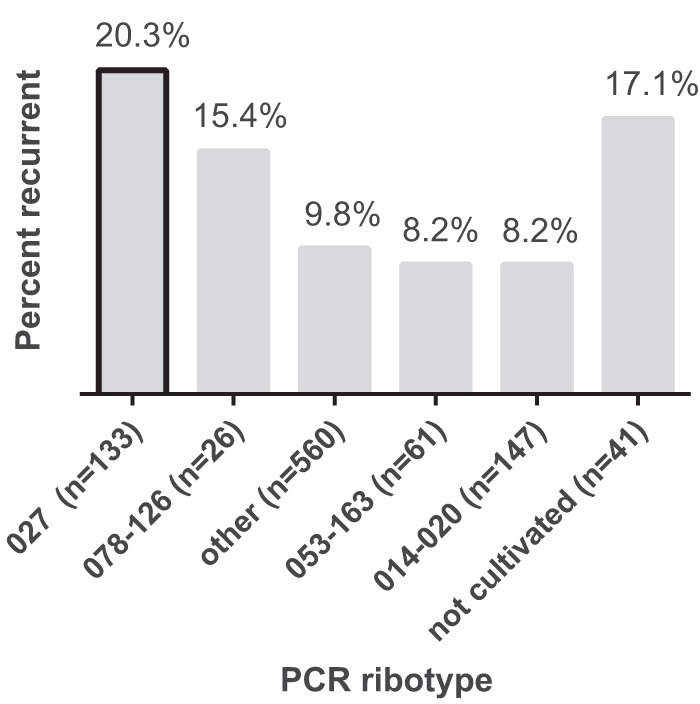
PCR ribotype of index CDI episode and subsequent recurrent CDI risk. Infection with ribotype 027 carries the highest risk of recurrent CDI.

Selected results from the baseline patient characteristics and outcomes are summarized in Table 1. In total, 899 patients with 968 index episodes of CDI were included, with 110 (11.4%) developing recurrent CDI. Notably, our cohort had slightly more women (54.3%) and was predominantly white (85.2%). The majority of patients were on proton pump inhibitors (PPIs) and were receiving concurrent antibiotics for an infection other than CDI and/or had hospital-associated CDI (HA-CDI). The breakdown of recurrent CDI by ribotype is shown in Fig. 1. We were able to culture and ribotype C. difficile from 927 (95.7%) stool samples. Among those, infection with ribotype 027 had the largest risk of recurrence (20.3%), followed by infection with ribotype 078-126 (15.4%). There were 79 (8.2%) deaths within 30 days of diagnosis.

**TABLE 1:** Selected baseline characteristics, outcomes, and unadjusted analysis versus recurrent CDI (968 index episodes; 110 recurrences)^a^

Variable	*n* (%) or mean ± SD	OR (95% CI)	*P*
Age (yrs)	57.1 (±18)	1 (0.99–1.01)	0.538
Female gender	526 (54.3)	1.41 (0.94–2.13)	0.096
White race	790 (81.6)	0.66 (0.4–1.07)	0.094
Charlson-Deyo score, unweighted	1.8 (±1.7)	1.08 (0.97–1.21)	0.181
Prior CDI	128 (13.2)	0.78 (0.42–1.47)	0.448
HA-CDI	702 (72.5)	0.52 (0.35–0.79)	0.002
Concurrent antibiotic use	645 (66.6)	1.25 (0.81–1.93)	0.313
Prior fluoroquinolone use	310 (32)	1.09 (0.71–1.65)	0.700
PPI use	660 (68.2)	1.28 (0.82–1.99)	0.278
Fever	206 (21.3)	0.91 (0.56–1.47)	0.687
Systolic blood pressure (mm Hg)	99.1 (±19.4)	1 (0.99–1.01)	0.692
Mechanical ventilation	172 (17.8)	0.59 (0.32–1.08)	0.086
Serum sodium (mmol/liter)	137 (±4.3)	0.97 (0.92–1.01)	0.133
Serum creatinine (mg/dl)	1.6 (±1.8)	0.998 (0.9–1.11)	0.969
Serum albumin (g/dl)	3.2 (±0.6)	1.01 (0.73–1.39)	0.959
Total serum bilirubin (mg/dl)	1.5 (±3.6)	1.05 (1–1.1)	0.034
Serum WBC >15,000 cells/mm^3^	286 (29.6)	0.997 (0.98–1.01)	0.729
Serum hemoglobin (g/dl)	9.5 (±2)	0.985 (0.89–1.09)	0.763
Serum platelets (1,000 cells/mm^3^)	256 (±190)	1 (0.999–1)	0.776
Ribotype (reference level: other ribotypes)			
Ribotype 014-020	147 (15.1)	0.82 (0.43–1.57)	0.542
Ribotype 027	133 (13.7)	2.34 (1.41–3.88)	0.001
Ribotype 053-163	61 (6.3)	0.82 (0.32–2.13)	0.684
Ribotype 078-126	26 (2.6)	1.67 (0.56–5.02)	0.362
Other ribotypes	560 (57.9)	NA	NA
Inability to cultivate C. difficile	41 (4.2)	1.65 (0.66–3.6)	0.244
Detectable stool toxin(s) A/B by EIA	354 (36.6)	1.87 (1.25–2.79)	0.002
*C_T_*	34 (±4)	0.94 (0.87–1.01)	0.099
Abnormal abdominal imaging	247 (25.5)	0.84 (0.53–1.35)	0.476
Severe CDI	324 (33.4)	1.2 (0.79–1.81)	0.393
Complicated CDI	322 (33.2)	0.9 (0.6–1.34)	0.604
30-day ICU admission	120 (12.4)	1.34 (0.77–2.34)	0.303
30-day mortality	79 (8.2)	NA	NA

a *C*omorbidities with nonsignificant *P* values not shown in this table: immunosuppression, AIDS, lymphoma, solid-organ tumor, metastatic cancer, obesity, liver disease, peptic ulcer disease, hypertension, prior myocardial infarction, congestive heart failure, peripheral vascular disease, prior stroke, dementia, chronic pulmonary disease, rheumatologic disorder, diabetes, chronic kidney disease, and depression. CDI, Clostridium difficile infection; CI, confidence interval; *C*_*T*_, PCR cycle threshold; EIA, enzyme immunoassay; HA, hospital associated; ICU, intensive care unit; NA, not applicable; OR, odds ratio; PPI, proton pump inhibitor; WBC, white blood cell count.

In the initial bivariable analysis, only HA-CDI, serum bilirubin, diagnosis of CDI by presence of toxin(s) A/B by enzyme immunoassay (EIA), and infection with ribotype 027 were significantly associated with recurrent CDI (Table 1). Notably, prior CDI, index CDI episode severity, age, PPIs, and concurrent antibiotic use were not associated with recurrent CDI. No other ribotype was associated with recurrence. Multiple variables were significantly associated with ribotype 027 (Table 2), and these were considered for adjustment in the multivariable model.

**TABLE 2:** Selected results from simple logistic regression of predictors versus infection with ribotype 027^a^

Variable	OR (95% CI)	*P*
Age (yrs)	1.03 (1.02–1.05)	<0.001
Charlson-Deyo score, unweighted	1.26 (1.14–1.39)	<0.001
HA-CDI	0.52 (0.35–0.76)	0.001
Solid-organ tumor	2.03 (1.31–3.14)	0.002
Prior myocardial infarction	2.08 (1.28–3.38)	0.003
Congestive heart failure	2.01 (1.26–3.21)	0.004
Diabetes mellitus	1.63 (1.1–2.41)	0.016
Chronic kidney disease	1.7 (1.15–2.52)	0.008
Concurrent antibiotic use	1.75 (1.14–2.67)	0.010
PPI use	1.52 (0.997–2.31)	0.052
Mechanical ventilation	0.54 (0.28–1.01)	0.055
Serum albumin (g/dl)	0.52 (0.38–0.71)	<0.001
Serum WBC >15,000 cells/mm^3^	2.05 (1.4–2.98)	<0.001
Detectable stool toxin(s) A/B by EIA	3.29 (2.25–4.81)	<0.001
30-day ICU admission	2.07 (1.28–3.34)	0.003

a CI, confidence interval; EIA, enzyme immunoassay; HA, hospital associated; ICU, intensive care unit; OR, odds ratio; PPI, proton pump inhibitor; WBC, white blood cell count.

### Multivariable modeling.

**FIG 2:**
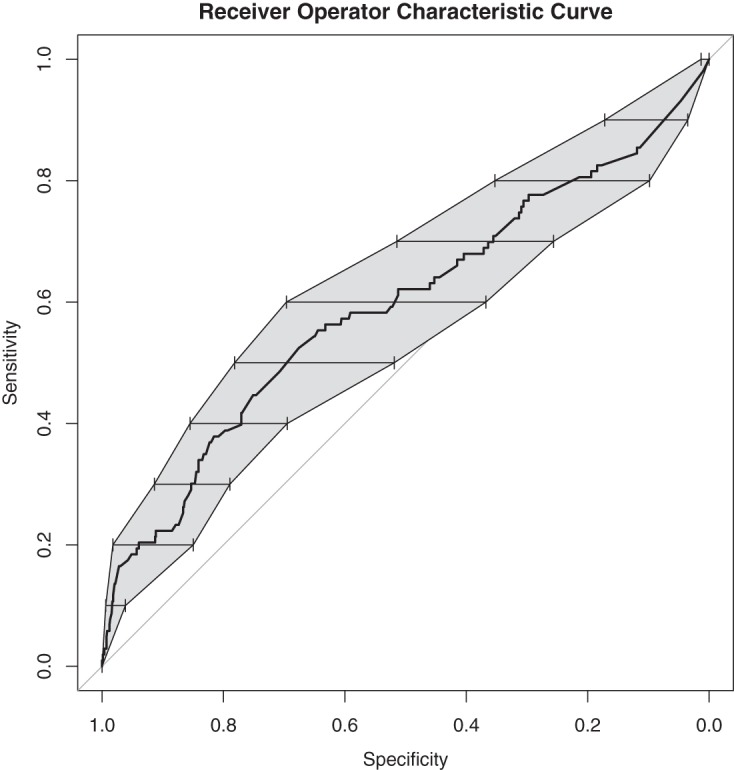
Receiver operator characteristic curve for the final multivariable model of recurrent CDI. The shaded area and bars represent bootstrapped confidence intervals (CIs) for specificity at each of the respective levels of sensitivity. The area under the curve (AUC) was 0.59 (95% CI, 0.53 to 0.66). The portion of highest specificity (left side of the curve) is primarily responsible for raising the AUC above 0.5.

The final multivariable model is shown in Table 3, which was arrived at by both the forward and backward selection procedures described in Materials and Methods. Interactions between variables in the final model were tested, and none of the interactions were significant. Thus, after adjustment for HA-CDI and serum bilirubin, ribotype 027 remained a significant independent predictor of recurrent CDI (odds ratio [OR], 2.17; 95% confidence interval [CI], 1.33 to 3.56; *P* = 0.002). Adding back into the model several variables demonstrated to associate with recurrence in other studies, specifically, age, PPI use, and concurrent antibiotics, did not affect this relationship between ribotype 027 and recurrence (data not shown). Additionally, adding back in other potential confounders associated with ribotype 027 on bivariable analysis (Table 2) did not change the point estimates or the significance of the association between ribotype 027 and recurrence (data not shown). We further explored HA-CDI, since the inverse association with recurrence was unexpected. Variables common among hospitalized, sick patients were associated with HA-CDI (obesity, congestive heart failure, and renal disease), but none of these affected the inverse association between HA-CDI and rCDI and were not included for adjustment in the models (data not shown). When patients who had died within 30 days were excluded from the model, the association between ribotype 027 and recurrent CDI remained significant (OR, 2.45; 95% CI, 1.47 to 4.09; *P* = 0.001). The Hosmer-Lemeshow test did not suggest poor model fit (*P* = 0.172). The receiver operator characteristic (ROC) curve for the model is shown in Fig. 2. As suggested by the curve’s bootstrapped confidence intervals crossing the 50% line and the AUC value of 0.59, the model’s predictive ability was poor overall.

**TABLE 3:** Final multivariable model of recurrent CDI

Variable	OR (95% CI)	*P*
HA-CDI	0.53 (0.35–0.82)	0.004
Serum bilirubin (mg/dl)	1.05 (1.01–1.1)	0.024
Ribotype 027	2.17 (1.33–3.56)	0.002

## DISCUSSION

The results from this study support the hypothesis that the infecting strain of the index CDI episode contributes to the risk of subsequent recurrent CDI. Specifically, the data identify infection with ribotype 027 as an independent predictor of recurrence even when adjusted for potential confounders. This result is in line with prior studies conducted from 2001 to 2017 that have also shown such an association. Three of these were much smaller studies (20–22), and two larger ones of comparable size to the current study were conducted outside the United States (18, 19). Thus, our results bolster the claim that ribotype 027 infection is associated with increased risk of rCDI. Notable strengths of our study included its large size, use of a validated ribotyping protocol, and careful attention to variable construction and analysis.

Knowledge of the infecting strain could be an important tool for clinicians who aim to stratify patients with respect to risk for recurrent CDI. However, our model with an AUC value of only 0.59 is comparable in performance to models reported from prior studies (11). This suggests that much work needs to be done to improve model performance, possibly through incorporation of additional, novel biomarkers. It is unlikely that ribotype information alone would have much clinical utility, but it might still be useful in an integrative model alongside clinical variables, microbiological factors, and host-level biomarkers. We utilized PCR ribotyping to distinguish strains, but approaches such as whole-genome sequencing may reveal other genome-derived biomarkers of adverse outcomes such as severity and recurrence. Evaluations of the performance of published models have not yet shown an AUC value above 90%, the level where reliable allocations of expensive and/or invasive treatments such as those employing fidaxomicin, monoclonal antibodies, or FMT can be made (25, 26). Furthermore, ribotyping may be too coarse a typing method to lead one to general conclusions, as strains that are categorized as ribotype 027 strains can have variable *in vitro* characteristics, such as sporulation (27). Finally, host factors also influence the risk of recurrent CDI and the relative importance of these versus strain needs to be determined.

There are notable results from our study that differ from results from prior studies. We identified associations with recurrence that not previously been described, including serum bilirubin and HA-CDI, and these need to be independently validated. HA-CDI in particular was inversely associated with recurrence, and we could not explain this within the scope of the current study. One possibility is that those with HA-CDI were less prone to CDI and recurrence for other reasons and developed CDI only with the increased colonization pressure of the hospital setting. This would imply they were less likely to have a history of prior CDI outside the current hospital admission. This was true in our study, where only 10.7% of patients with a current index HA-CDI episode had had a prior CDI episode(s), compared to 19.9% of those without a current HA-CDI episode having had one or more prior CDI episodes (*P* < 0.001). However, adjustment for prior CDI did not affect the inverse relationship between HA-CDI and recurrence (OR for recurrence with HA-CDI, 0.51; 95% CI, 0.34 to 0.77; *P* = 0.001). As before, excluding the patients who died, a possible source of bias against increased recurrence, did not significantly alter these results (OR, 0.59; 95% CI, 0.29 to 1.1; *P* = 0.002).

It is also notable that our study failed to validate the contention that age, PPI use, or concurrent antibiotic use is associated with recurrence. Prior studies have consistently demonstrated such associations (28). Age is also associated with ribotype 027 infection, although this does not explain the lack of association seen in our study (29). Although it is not clear why age did not associate with recurrence in our study, PPI and concurrent antibiotic use were highly prevalent in our cohort and, thus, the discriminatory ability of statistical tests may have been compromised.

Our study was also limited by its having been conducted at a single center, by the use of retrospective data extraction, and by the high likelihood of misclassification bias. The high risk of misclassification bias occurred because our hospital is a tertiary care referral center. There is a probable failure to detect recurrence occurring in referral center areas that are not served by our hospital’s microbiology laboratory. That is, inpatients living further away who had developed HA-CDI and had then experienced disease recurrence were less likely to have had the recurrence diagnosed at our center, thus resulting in an erroneous protective association with rCDI. This is a threat to the internal validity of our study’s results regarding HA-CDI and rCDI, and the results thus require external validation. Our center uses multistep testing in the clinical microbiology laboratory; thus, many subjects were diagnosed by PCR and not toxin detection. The optimal testing methodology for CDI and the possible association of toxin detection with increased mortality are hotly debated without a clear consensus among experts or guidelines (30), but this could have influenced our results. Additionally, only one isolate from each sample was recovered, and prior studies have shown that mixed infections with different C. difficile strains can occur (31). Finally, we did not have isolates from the recurrent episodes and thus could not determine if the recurrence involved the same strain, though that determination is beyond the scope of this study, which was focused on the risk of recurrence based on the infecting strain.

### Conclusions.

Overall, our study data suggest that infection with PCR ribotype 027 during a nonrecurrent index episode of CDI is independently associated with subsequent recurrent CDI. The overall predictive ability of this finding is poor; thus, novel biomarkers for recurrent CDI should be sought to aid clinicians. Since the infecting strain has prognostic implications but "ribotype" is a rather coarse classification, the increased resolution of whole-genome sequencing means that it is a potential tool for future investigations that may uncover other useful, genome-derived biomarkers that improve the performance of predictive models for recurrent CDI.

## MATERIALS AND METHODS

### Patients and clinical data.

The University of Michigan Institutional Review Board approved this study. We selected subjects for inclusion from a previously described cohort of 981 patients (32). Briefly, in that cohort, stool samples from nonpregnant patients that were submitted to the clinical microbiology laboratory and that tested positive for presence of toxigenic C. difficile (testing details below) were prospectively and consecutively included between October 2010 and January 2013. Initial laboratory testing was performed at the discretion of the inpatient team. From that cohort, we selected only patients with a primary, nonrecurrent (i.e., index) episode of CDI, i.e., patients with whom the episode had not occurred within 8 weeks of a prior episode (33). However, we included those with a history of CDI at >8 weeks prior to the current episode. We defined recurrent CDI as positive stool testing for toxigenic C. difficile >2 weeks but ≤8 weeks from the index episode as suggested by the Centers for Disease Control and Prevention (33), again with testing driven by the clinical team. All testing was done by the University of Michigan Clinical Microbiology Laboratory.

Data were extracted from the chart as previously described (32). Briefly, we classified CDI cases as hospital-associated (HA-CDI) cases if symptoms developed >72 h after admission (33). We also collected demographics, medical history, unweighted Charlson-Deyo comorbidity scores (34), vital signs, and laboratory test results. Since our goal was to identify factors available to a clinician at the time of diagnosis of the index episode, we limited our search for the above variables to ±48 h from diagnosis. Severe CDI was defined as a white blood cell count (WBC) of ≥15,000 cells/mm^3^ and/or an elevation of serum creatinine ≥1.5 times the premorbid value (35). Complicated CDI was defined as the presence of hypotension, shock, ileus, or megacolon (35). We separately assessed whether abdominal radiographic imaging was abnormal (evidence of colitis, colonic thickening, ileus, distension, perforation, or peritonitis).

### Microbiology.

The clinical microbiology laboratory tested stool samples for toxigenic C. difficile with a two-step algorithm. The initial step used the C. Diff Quik Check Complete test for C. difficile glutamate dehydrogenase (GDH) and toxin A or B by the use of an enzyme immunoassay (Techlab, Inc., Blacksburg, VA). All initial step results that were discordant (GDH positive and toxin negative [GDH^+^/toxin^−^] or GDH^−^/toxin^+^) were reanalyzed (reflexed) using a real-time PCR for the *tcdB* gene and the GeneOhm Cdiff assay (BD, Franklin Lakes, NJ) run on a Cepheid SmartCycler system (Cepheid, Sunnyvale, CA). Where available, PCR cycle threshold (*C*_*T*_) values were obtained through query of the SmartCycler database. Confirmation of positive tests was attempted by anaerobic culture on taurocholate-cycloserine-cefoxitin-fructose agar at 37°C, and isolates were ribotyped using a high-throughput, fluorescent PCR ribotyping protocol previously validated at multiple sites and described elsewhere (36).

### Statistical analysis.

All analyses were conducted in R version 3.3.1 (R Foundation for Statistical Computing, Vienna, Austria), and a two-tailed *P* value of <0.05 was considered significant for all analyses. The strategy for handling variable constructions and missing values, including use of imputation with the R package *missForest* version 1.4 (37), was previously described (32). Bivariable relationships were assessed using simple logistic regression for the outcome of recurrent CDI but also for comparisons between ribotype 027 and other variables, since only ribotype 027 was significantly associated with recurrence (see the Results section). To build multivariable models testing our hypothesis that ribotype was associated with recurrence, we started with a base model that included "ribotype" as the sole predictor. We subsequently built adjusted models via stepwise addition and included variables that had a likelihood ratio test with *P* values of <0.05. Special attention was paid to any variables that were significant with respect to the initial bivariable, unadjusted analysis performed either with recurrence or with ribotype 027. We also performed backwards elimination starting with a full model and compared the final models arrived at by both strategies. Finally, the analysis was repeated by excluding those patients who had died within 30 days of diagnosis to assess if inclusion of these patients introduced bias. The final model’s fit was further assessed by the Hosmer-Lemeshow test (S. R. Lele, J. L. Keim, and P. Solymos, R package ResourceSelection, https://cran.r-project.org/web/packages/ResourceSelection/ResourceSelection.pdf) and by calculating the area under the receiver operator characteristic (AUROC) curve. To obtain 95% confidence intervals for the ROC curve, bootstrapping using 10,000 replicates was employed with the R package *pROC* (38). Interactions between variables in the final model were assessed and included if significant (*P* < 0.05).
